# Latin American Obstetrics and Gynecology. What is Up with the Journals?

**DOI:** 10.1055/s-0041-1740280

**Published:** 2022-02-25

**Authors:** Mario Arturo González-Mariño

**Affiliations:** 1 Department of Obstetrics and Gynecology, Faculty of Medicine, Universidad Nacional de Colombia, Bogotá D.C., Colombia

Dear Editor,

The Scimago Journal and Country Rank 1 measures and classifies the journals and country scientific indicators from the information contained in the Scopus database. 1 2 This portal uses several indicators such as the SJR, defined as “a measure of the impact, influence, or prestige of the journal. It expresses the average number of weighted citations received in the selected year by the documents published in the journal in the 3 previous years”, and the H-index, “the number of articles of the journal (h) that have received at least h citations over the whole period”. 1

The rank of Obstetrics and Gynecology journals from Latin America and their settings are shown in the tables. The first in the SJR rank is the “Jornal Brasileiro de Reprodução Assistida” from Brazil, placed 8,257 in the rank of 25,232 world journal titles on “all subject areas” and 86 of 181 Obstetrics and Gynecology world journals (it is the only journal in Latin America classified in the Q2 SJR Quartile), followed by the “Revista Brasileira de Ginecologia e Obstetrícia” (Q3), 11,399 in the whole rank and 104 in the Obstetrics and Gynecology world journals, but the first in the H-index within this group in Latin America in the year 2020.

Latin American Obstetrics and Gynecology journals classified in the Scimago Journal Rankings are few, and those included are not much cited. Several reasons contribute to this situation, such as that the best quality articles are often sent to international journals, the scarce training in research during residency in these countries, the limitations of the authors and journals for the publication of articles in English to find better visibility, and the difficulty in financing the journals, which in some cases have led to the suspension of some issues or even of the journal itself or to compromise editorial independence when they need to be financed by the pharmaceutical industry. 3 However, the preservation and strengthening of these journals are important for Latin America because they serve as a means of dissemination for its researchers, and, occasionally, they focus on issues of local or regional importance. Obstetrics and Gynecology journals in Latin America should seek to improve the quality, visibility, and access of their publications to try to generate a greater number of citations ( Charts 1 to 5 are available as Supplementary material ). 1
