# Supplementary material for: Latin American Obstetrics and Gynecology. What is Up with the Journals?
Source: Rev Bras Ginecol Obstet. 2022 Feb 25;44(2):201. doi: 10.1055/s-0041-1740280 (PMC9948056; doi:10.1055/s-0041-1740280)
Supplement: Supplementary file 1 — Supplementary Material [file 10-1055-s-0041-1740280-s210232.pdf]

Chart 1 Scimago journal rankings of Obstetrics and Gynecology in Latin America, 2020

Rank	Title	SJR	SJR Quartile	H index
1	Jornal Brasileiro de Reprodução Assistida	0.596	Q2	14
2	Revista Brasileira de Ginecologia e Obstetrícia	0.413	Q3	23
3	Revista Brasileira de Saúde Materno Infantil	0.248	Q3	20
4	Revista Cubana de Obstetricia y Ginecologia	0.158	Q3	9
5	Revista Colombiana de Obstetricia y Ginecologia	0.139	Q4	8
6	Revista Chilena de Obstetricia y Ginecologia	0.131	Q4	11
7	Ginecologia y Obstetricia de Mexico	0.126	Q4	16
8	Reprodução e Climatério	0.122	Q4	5
9	Revista de Obstetricia y Ginecologia de Venezuela	0.11	Q4	8

Chart 2 Journals of Obstetrics and Gynecology in Latin America, 2020. Indexes and the journals' countries

Title	Total Docs. (2020)	Total Docs. (3years)	Total Refs.	Total Cites (3years)	Citable Docs. (3years)	Cites/ Doc. (2years)	Ref. / Doc.	Country
Jornal Brasileiro de Reprodução Assistida	90	203	2685	738	186	3	29.83	Brazil
Revista Brasileira de Ginecologia e Obstetrícia	133	358	3582	811	306	1.89	26.93	Brazil
Revista Brasileira de Saúde Materno Infantil	62	157	1543	148	143	0.47	24.89	Brazil
Revista Cubana de Obstetricia y Ginecologia	41	165	955	64	161	0.35	23.29	Cuba
Revista Colombiana de Obstetricia y Ginecologia	30	85	877	40	71	0.35	29.23	Colombia
Revista Chilena de Obstetricia y Ginecologia	84	206	2084	60	186	0.23	24.81	Chile
Ginecologia y Obstetricia de Mexico	130	307	2875	80	271	0.21	22.12	Mexico
Reprodução e Climatério	0	26	0	18	26	0	0	Brazil
Revista de Obstetricia y Ginecologia de Venezuela	20	96	540	8	87	0.07	27	Venezuela

 © 2022. Federação Brasileira de Ginecologia e Obstetrícia. All rights reserved.

Chart 3 Scimago country rankings of Obstetrics and Gynecology in Latin America and indexes, 1996–2020

Rank	Country	Documents	Citable documents	Citations	Self-citations	Citations pd	H index
1	Brazil	9338	8651	113050	26625	12.11	98
2	Mexico	3387	3277	27483	4114	8.11	63
3	Chile	2056	1941	37769	4120	18.37	86
4	Argentina	1598	1452	31395	3917	19.65	75
5	Colombia	1204	1123	9051	1064	7.52	43
6	Cuba	915	897	3201	827	3.5	25
7	Venezuela	670	644	3289	391	4.91	28
8	Peru	451	415	5209	522	11.55	36
9	Ecuador	307	288	5169	702	16.84	38
10	Uruguay	199	177	3319	210	16.68	26
11	Guatemala	142	133	1878	301	13.23	24
12	Jamaica	138	126	1667	131	12.08	23
13	Puerto Rico	121	102	1794	126	14.83	24
14	Panama	108	98	1197	74	11.08	20
15	Dominican Republic	97	97	2363	157	24.36	29
16	Paraguay	56	52	713	27	12.73	13
17	Trinidad and Tobago	53	50	537	5	10.13	10
18	Costa Rica	52	46	818	33	15.73	13
19	Haïti	48	42	665	66	13.85	12
20	Bolivia	47	45	879	54	18.7	15

Chart 4 Scimago country rankings of Obstetrics and Gynecology in Latin America, 2020

Rank	Country	Documents	Citable documents	Citations	Self-citations	Citations per document	H index
1	Brazil	754	663	513	118	0.68	98
2	Mexico	230	216	104	18	0.45	63
3	Colombia	153	140	192	16	1.25	43
4	Chile	148	137	163	12	1.1	86
5	Argentina	110	92	134	16	1.22	75
6	Peru	55	52	157	2	2.85	36
7	Ecuador	43	39	35	4	0.81	38
8	Cuba	35	35	1	0	0.03	25
9	Guatemala	29	26	21	1	0.72	24
10	Venezuela	18	17	11	1	0.61	28
11	Uruguay	17	14	19	0	1.12	26
12	Paraguay	15	14	18	2	1.2	13
13	Panama	12	11	3	0	0.25	20
14	Jamaica	11	11	7	0	0.64	23
15	Bolivia	9	8	9	2	1	15
16	Puerto Rico	8	7	5	1	0.63	24
17	Costa Rica	5	4	1	0	0.2	13
18	Grenada	5	4	2	1	0.4	5
19	Nicaragua	4	4	0	0	0	13
20	French Guiana	4	4	2	0	0.5	9

 © 2022. Federação Brasileira de Ginecologia e Obstetrícia. All rights reserved.

Chart 5 Scimago's research ranking of universities in Latin America, 2020

Rank	Global Rank	Institution	Country
1	61	Universidade de São Paulo	Brazil
2	302	Universidad Nacional Autónoma de México	Mexico
3	336	Universidade Estadual Paulista Júlio de Mesquita Filho	Brazil
4	379	Universidade Estadual de Campinas	Brazil
5	407	Universidade Federal do Rio de Janeiro	Brazil
6	445	Universidade Federal de Minas Gerais	Brazil
7	448	Universidade Federal do Rio Grande do Sul	Brazil
8	473	Universidad de Chile	Chile
9	489	Pontificia Universidad Católica de Chile	Chile
10	503	Universidad de Buenos Aires	Argentina
11	528	Universidade Federal de São Paulo	Brazil
12	570	Universidade Federal de Santa Catarina	Brazil
13	577	Universidade Federal do Paraná	Brazil
14	593	Universidad Nacional de Colombia	Colombia
14	593	Universidad de Puerto Rico, Ciencias Medicas	Puerto Rico
15	609	Universidade de Brasilia	Brazil
15	609	Universidad de Puerto Rico	Puerto Rico
16	633	Universidade Federal de Viçosa	Brazil
17	636	Universidade Federal de São Carlos	Brazil
18	642	Universidade do Extremo Sul Catarinense	Brazil
19	644	Universidade Federal do Ceará	Brazil
20	646	Universidade Federal de Santa Maria	Brazil

 © 2022. Federação Brasileira de Ginecologia e Obstetrícia. All rights reserved.
